# The effect of biologics in lung function and quality of life of patients with united airways disease: A systematic review

**DOI:** 10.1016/j.jacig.2023.100174

**Published:** 2023-09-28

**Authors:** Javier Domínguez-Ortega, Joaquim Mullol, Francisco Javier Álvarez Gutiérrez, Celia Miguel-Blanco, Jose Antonio Castillo, Jose María Olaguibel, Marina Blanco-Aparicio

**Affiliations:** a Department of Allergy, La Paz University Hospital, Institute for Health Research (IdiPAZ), CIBER of Respiratory Diseases (CIBERES), Madrid, Spain; b Rhinology Unit & Smell Clinic, ENT Department, Hospital Clinic, Universitat de Barcelona, IDIBAPS, CIBERES, Barcelona, Spain; c Medical and Surgical Unit for Respiratory Diseases, Hospital Virgen del Rocío, Sevilla, Spain; d Medical Statistics Consulting S.L., Valencia, Spain; e Department of Respiratory Medicine, Hospital Universitari Dexeus, Barcelona, Spain; f Department of Allergy, Hospital Universitario de Navarra, CIBER of Respiratory Diseases (CIBERES), Navarra, Spain; g Department of Respiratory Medicine, Complexo Hospitalario Universitario de A Coruña A Coruña Spain

**Keywords:** Asthma, chronic rhinosinusitis with nasal polyps, lung function, quality of life, united airways disease

## Abstract

**Background:**

Increasing evidence supports the united airway disease concept for the management of upper and lower respiratory tract diseases, particularly in patients with asthma and chronic rhinosinusitis with nasal polyps (CRSwNP). However, evidence for a combined approach in asthma and CRSwNP is scarce.

**Objective:**

In this systematic review, we focused on the role of biologics in the lung function and quality of life in patients with severe asthma and CRSwNP.

**Methods:**

We conducted a systematic search of 3 electronic databases using 2 search strategies to identify studies published from January 2010 to March 2022. Quality assessment was performed with the Critical Appraisal Skills Programme.

**Results:**

Of 1030 studies identified, 48 original studies reporting data of benralizumab (12), dupilumab (14), mepolizumab (10), omalizumab (13), and reslizumab (2) were analyzed. Primary diagnosis was mostly asthma or CRSwNP, with only 15 studies, mainly observational, performed in populations with united airway disease. In total, 18 studies reported data on quality of life (mostly 22-item Sino-Nasal Outcome Test score), 8 on lung function (mostly FEV_1_), and 22 on both outcomes. Significant FEV_1_ and 22-item Sino-Nasal Outcome Test score improvements were consistently observed after 24-week treatment, and thereafter, mostly in real-world studies that included variable proportions of patients with asthma/CRSwNP.

**Conclusions:**

The use of biologics in patients with severe asthma and CRSwNP was overall associated with significant improvements in lung function and quality of life. However, we observed a high heterogeneity of populations and outcome measurements across studies. Notwithstanding the need of larger studies, our results reinforce the joint management of asthma and CRSwNP as united airway disease in clinical practice.

Upper and lower respiratory tract diseases have traditionally been managed as independent entities in clinical practice. Nowadays, increasing evidence supports a paradigm shift toward united airways disease (UAD),^1^, ^2^, ^3^, ^4^ a concept based on the common pathophysiological and immunologic mechanisms that underlie certain respiratory diseases,^5^^,^^6^ such as the eosinophilic airway inflammation associated with T_H_2 cytokines (IL-4, IL-5, and IL-13) and/or IgE.^1^^,^^7^ The UAD concept is particularly relevant in the context of multimorbidity due to severe asthma and chronic rhinosinusitis with nasal polyps (CRSwNP), a clinical scenario that is particularly common, severe, and difficult to treat. The prevalence rate of asthma in patients with CRSwNP is estimated to be up to 60% to 70%, whereas severe asthma is associated with nasal polyps in more than 70% of cases.^1^^,^^7^^,^^8^ Multimorbidity is associated with worse outcomes and more severe disease,^7^^,^^8^ leading to an increased use of systemic corticosteroids in both diseases. Moreover, approximately 10% of patients with CRSwNP present aspirin-exacerbated respiratory disease (AERD) or nonsteroidal anti-inflammatory drug–exacerbated respiratory disease.^1^^,^^9^

The symptomatology of UAD refers to that described for both CRSwNP and asthma, but its combination often results in a higher symptom burden, worse asthma control, and poorer lung function and quality of life (QOL).^10^ Some studies have reported higher rates of nasal polyps recurrence^11^ and asthma exacerbation,^12^ possibly due to increased airway obstruction and eosinophilic inflammation. Therapeutic approaches in patients with UAD are mainly focused on minimizing the dose of systemic corticosteroids and increasing the use of biologics.^13^, ^14^, ^15^ Numerous studies have consistently reported the clinical benefit of biologics in upper and lower respiratory tract diseases. In fact, significant improvements in asthma and sinonasal outcomes and a positive impact on QOL^16^, ^17^, ^18^ with dupilumab, omalizumab, and mepolizumab have led to the approval of these drugs in the treatment of asthma and CRSwNP. Reslizumab and benralizumab, which are currently approved for asthma,^15^^,^^19^ have also shown promising results in patients with CRSwNP.^20^^,^^21^

Applying a multidisciplinary, UAD-based approach to the management of these patients is still a challenge and an unmet need. Given the lack of recommendations for joint management in current clinical practice guidelines,^13^^,^^15^^,^^19^ an evidence-based approach could help decision-making processes. However, the considerable heterogeneity among clinical trials, *post hoc* analyses, and real-world studies performed in patients with UAD makes it difficult to compare data and findings.^2^ Moreover, studies in CRSwNP rarely evaluate asthma severity, and few asthma trials take severity of CRSwNP into consideration, hampering the evaluation of treatment response in these populations. With this background, we performed this systematic review to explore and analyze the role of biologics in UAD, specifically, their effect on lung function and QOL in patients with severe asthma and CRSwNP.

## Methods

This systematic review follows the recommendations of the PRISMA guidelines^22^ (see Table E1 in this article’s Online Repository at www.jaci-global.org) and the Cochrane handbook for systematic reviews.^23^ The search protocol was registered in the international prospective register of systematic reviews, PROSPERO (CRD42022318548).

### Eligibility criteria

Systematic reviews with or without meta-analyses, randomized clinical trials (RCTs) and nonrandomized trials, *post hoc* studies of RCTs, and observational studies were included. Case reports and series, narrative reviews, letters to the editor, expert consensus, and preclinical studies were excluded. Only studies reporting on lung function and/or QOL in patients with asthma and CRSwNP and/or AERD who were treated with biologics were considered for inclusion. Lung function outcomes included FEV_1_, percentage of FEV_1_ predicted, prebronchodilator FEV_1_, postbronchodilator FEV_1_, forced vital capacity, FEV_1_/forced vital capacity, forced midexpiratory flow, and peak expiratory flow. QOL assessments determined by the Asthma Quality of Life Questionnaire (AQLQ), 22-item Sino-Nasal Outcome Test (SNOT-22), mini-AQLQ, St. George’s Respiratory questionnaire, short-form 36 questionnaire, and Rhinosinusitis Outcome Measure were included.

### Search strategy

The literature search was performed in PubMed/MEDLINE, Scopus, and Web of Science databases; studies in English and Spanish published between January 2010 and March 2022 were considered. A research question was formulated using the PICO structure (P, patient; I, intervention; C, comparator; O, outcome), and strategies were subsequently defined according to expert advice. Two search strategies were developed, on the basis of the following research question: “What are the clinical outcomes in terms of lung function and/or QOL in patients with UAD (asthma and CRSwNP) who receive biologics?” The interventions included all available biologics (anti-IgE [omalizumab], anti–IL-4Rα [dupilumab], anti–IL-5 [mepolizumab and reslizumab], and anti–IL-5Rα [benralizumab] mAbs), and outcomes related to QOL and/or lung function were included. Search strategies were adapted for each database (see Table E2 in this article’s Online Repository at www.jaci-global.org).

### Study selection and data collection

The results were screened by 2 independent reviewers. Following the predefined inclusion and exclusion criteria, publications were first selected on the basis of title/abstract, and then after full-text reading. Data on study design, patient characteristics, main outcomes, and additional findings were extracted from the studies and uploaded by one of the reviewers to a standardized Microsoft Excel template, which was then double-checked and validated by the second reviewer.

### Methodological quality assessment

We performed a quality assessment of the selected studies using the Critical Appraisal Skills Programme checklists (https://casp-uk.net/casp-tools-checklists/). Study design, methodology, outcomes, and results were evaluated as described in the specific checklists.

### Data synthesis

Study outcomes, specifically those related to lung function and QOL, are presented in tables by biologics. Baseline, posttreatment, and change from baseline values from independent studies are indicated, if available, in the tables. Because of the heterogeneous nature of the studies included in this systematic review, the data were synthesized descriptively.

## Results

**Fig 1:**
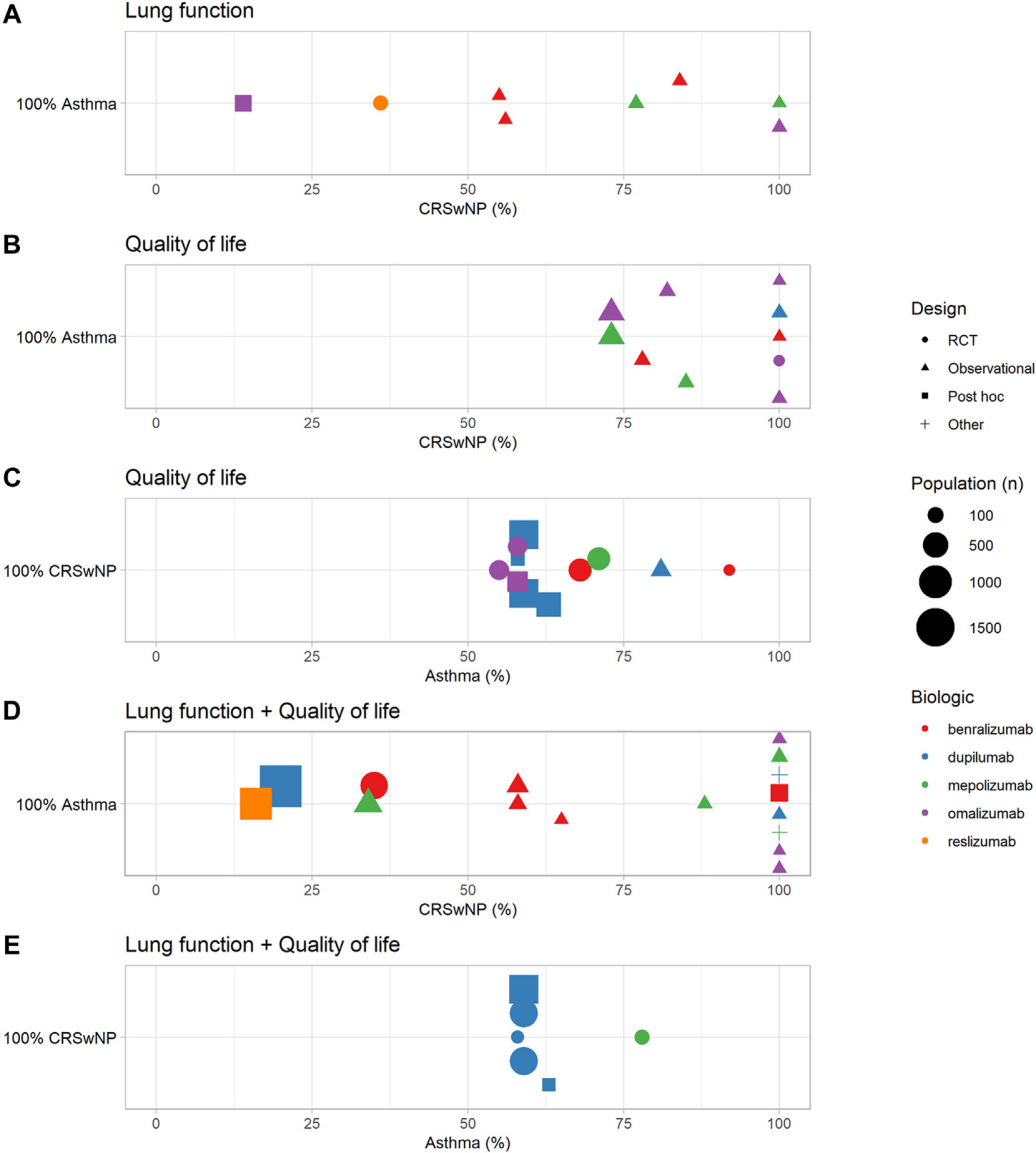
Characteristics of studies reporting on lung function (LF), QOL, or both. **A,** LF (all patients with asthma); **B,** QOL (all patients with asthma); **C,** QOL (all patients with CRSwNP); **D,** LF and QOL (all patients with asthma); **E,** LF and QOL (all patients with CRSwNP). Percentages on the X-axis indicate the proportion of patients with asthma or CRSwNP.

A total of 1030 studies were retrieved using the 2 search strategies (see Figs E1 and E2 in this article’s Online Repository at www.jaci-global.org). After duplicates had been removed, 613 studies were screened, of which 535 were excluded on the basis of title/abstract and 7 after full-text reading. Of the 71 articles selected for inclusion, 56 independent studies were identified. Of these, 48 were original studies and 8 were systematic reviews reporting data already identified in the original articles. Overall, methodological quality of most studies was high to moderately high (see Table E3 in this article’s Online Repository at www.jaci-global.org). Characteristics of the original publications are summarized in Fig 1.

### Benralizumab

Twelve studies reporting on benralizumab were found, of which 3 were RCTs,^20^^,^^24^^,^^25^ 1 was a *post hoc* analysis,^26^ and 8 were observational.^27^, ^28^, ^29^, ^30^, ^31^, ^32^, ^33^, ^34^ Of these, 10 studies considered asthma and 2 RCTs considered CRSwNP as the primary diagnosis of the study population (Table I). Only 1 observational prospective study^29^ and the *post hoc* analysis of the ANDHI trial^26^ reported data on 100% patients with UAD (ie, asthma + CRSwNP); the remaining studies included variable percentages of asthmatic patients in the population with CRSwNP (68%-83%)^20^^,^^25^ or patients with CRSwNP in the population with asthma (35%-91%).^24^^,^^27^^,^^28^^,^^30^, ^31^, ^32^, ^33^, ^34^ In total, 8 studies included lung function and 9 QOL outcomes; of these, 5 studies reported both.^24^^,^^26^^,^^27^^,^^30^^,^^33^

**Table I:** Studies reporting lung function and/or QOL with benralizumab

Reference	Study design	Population (n)	Asthma/CRSwNP	Change from baseline in lung function	Change from baseline in QOL
Bagnasco et al,^27^ 2020	OBS retrospective	Severe eosinophilic asthma (n = 59)	100%/58%	FEV_1_∗ +180 mL after 24 wk (NS)	SNOT-22 score −13.7 points after 24 wk (*P* = .004)
Bandi et al,^28^ 2020	OBS prospective	Patients with severe asthma (n = 40)	100%/78%	NA	SNOT-22 score∗† 56 (IQR, 33 to 70) at baseline to 24 (16-27) after 52 wk (*P* = .063)
Lombardo et al,^29^ 2020	OBS prospective	Severe eosinophilic allergic asthma (n = 10)	100%/100%	NA	SNOT-22 score∗ 61.1 ± 17.2 at baseline to 26.3 ± 19.7 after 24 wk (*P* < .001)
Matsuno et al,^30^ 2020	OBS retrospective	Severe eosinophilic asthma (n = 17)	100%/65%	FEV_1_∗‡ 55.3% ± 17.1% at baseline; improved significantly after 4,16, and 24 wk	AQLQ score∗ 5.3 ± 0.8 at baseline; improved significantly after 4, 16, 24, and 50 wk
Menzella et al,^32^ 2020	OBS retrospective	Severe eosinophilic asthma (refractory) (n = 18)	100%/55%	Pre-BD FEV_1_‡ 1.9 ± 0.8 L at baseline to 2.6 ± 1.1 L after 6 mo (*P* = .0004)	NA
Numata et al,^34^ 2020	OBS retrospective	Severe eosinophilic asthma (n = 24)	100%/77%-91%	FEV_1_‡ 2.0 ± 0.6 L at baseline to 2.1 ± 0.6 L after a median of 8 doses (*P* = .07)	NA
Harrison et al,^24^ 2021	RCT (ANDHI trial)	Severe eosinophilic asthma (n = 656)	100%/35%	FEV_1_ Baseline: 1.9 ± 0.6 L pre-BD/2.1 ± 0.7 L post-BD +160 mL (95% CI, 90 to 230) after 24 wk (*P* < .0001)	SNOT 22 score∗‡ Baseline: 51.5 ± 20.4 −8.9 (95% CI, −16.4 to −1.4) after 24 wk (*P* = .02)
Menzella et al,^31^ 2021	OBS retrospective	Severe eosinophilic asthma (n = 18)	100%/56%	FEV_1_‡ 1.9 ± 0.8 L at baseline to 2.6 ± 1.1 L after 26 wk (*P* = .0002) and 2.7 ± 1.1 L after 52 wk (*P* = .034)	NA
Nolasco et al,^33^ 2021	OBS retrospective	Severe eosinophilic asthma (n = 137)	100%/58%	FEV_1_‡ 1.7 L (IQR, 1.2 to 2.3) at baseline to 1.9 L (1.6 to 2.4) after 4 wk (*P* < .0001) and 2.1 L (1.7-2.6) after 24 wk (*P* < .0001)	SNOT-22 score 46 (IQR, 39.5 to 64.5) at baseline to 32 (19 to 46) after 24 wk (*P* < .0001)
Tversky et al,^25^ 2021	RCT	Severe CRSwNP (n = 24)	83%/100%	NA	SNOT-22 score Baseline: 57.6 ± 16.8 −19.2 points ± 2.6 after 20 wk (*P* < .001)
Bachert et al,^20^ 2022	RCT (OSTRO study)	CRSwNP (n = 413)	68%/100%	NA	SNOT-22 score Baseline: 63.9 ± 19.8 −5.2 (95% CI, −11.1 to 0.7) after 40 wk (*P* = .08) and −7.5 (−13.7 to −1.2) after 56 wk (*P* = .02)
Canonica et al,^26^ 2022	*Post hoc* (ANDHI trial)	Severe eosinophilic asthma (n = 153)	100%/100%	FEV_1_∗ Baseline: 1.7 ± 0.6 L pre-BD/2.1 ± 0.8 L post-BD +320 mL (95% CI, 60 to 470) after 24 wk (*P* < .0001)	SNOT-22 score∗‡ Baseline: 51.5 ± 20.4 −10.4 (95% CI, −19.0 to −1.9) after 24 wk (*P* = .018)

SNOT-22 scores range from 0 to 110. Higher SNOT-22 total-scores indicate worse symptoms.

*IQR*, Interquartile range; *N-ERD*, NSAID-exacerbated respiratory disease; *NS*, not significant; *NSAID*, nonsteroidal anti-inflammatory drug; *OBS*, observational study; *post-BD*, postbronchodilator; *pre-BD*, prebronchodilator.

∗ Results for patients with both asthma and CRSwNP are presented.

† Results from the 9 patients who received benralizumab are presented.

‡ This study reports other lung function and QOL parameters.

In patients with severe asthma, the SNOT-22 score decreased significantly after 24 weeks—by 34.8 points in an observational study and by 10.4 points in the *post hoc* analysis of the ANDHI trial.^26^^,^^29^ The baseline score was higher in the former study, which included only 10 patients.^29^ The *post hoc* analysis also reported a significant increase in FEV_1_ (320 mL) at week 24.^26^ Three studies assessed the impact of benralizumab on QOL in patients with severe asthma with CRSwNP (35%-78%). The SNOT-22 score decreased by 22.0 points after 52 weeks (observational study)^28^ and by 8.9 points after 24 weeks (RCT).^24^ In the ANDHI trial, FEV_1_ increased by 160 mL after 24 weeks,^24^ and an observational study showed significant FEV_1_ and AQLQ-score improvements after 4, 16, 24, and 50 weeks.^30^

Five observational studies reported the impact of benralizumab on lung function in patients with severe asthma with CRSwNP (58%-91%); 2 of them also evaluated the impact on QOL. In a population of 18 patients, FEV= significantly increased after 24 weeks (700 mL)^31^^,^^32^ and 52 weeks (800 mL).^31^ Likewise, FEV_1_ improvement was confirmed in a larger population at week 4 (200 mL) and week 24 (400 mL),^33^ whereas the increase was only numerical in other studies.^27^^,^^34^ The SNOT-22 score significantly decreased, by approximately 14 points (a minimal clinically important difference is defined as scores >8.9),^35^ after 24 weeks.^27^^,^^33^ Two RCTs reported the impact of benralizumab on QOL in patients with severe CRSwNP and asthma (68%-83%). A significant reduction in the SNOT-22 score was observed after 20 weeks (19.2 points)^25^ and 56 weeks (7.5 points).^20^ The baseline score and the number of patients were lower in the former study.

### Dupilumab

In total, 14 studies reported dupilumab data: 2 RCTs,^16^^,^^36^ 7 *post hoc* analyses,^37^, ^38^, ^39^, ^40^, ^41^, ^42^, ^43^ 1 RCT pooled analysis,^44^ 1 open-label trial,^45^ and 3 observational studies.^46^, ^47^, ^48^ Of these, 10 studies analyzed CRSwNP, 2 asthma, and 2 AERD as the primary diagnosis (Table II). One observational retrospective study reported data on 100% patients with UAD,^48^ whereas the remaining studies included 58% to 87% patients with asthma in the population with CRSwNP. The *post hoc* analysis of the LIBERTY ASTHMA QUEST included 20% patients with CRSwNP.^42^ Three studies reported data on patients with AERD.^43^^,^^45^^,^^46^ All studies included data on QOL, and 8 also reported lung function outcomes.^16^^,^^36^^,^^38^^,^^42^, ^43^, ^44^, ^45^^,^^48^

**Table II:** Studies reporting lung function and/or QOL with dupilumab

Reference	Study design	Population (n)	Asthma/CRSwNP	Change from baseline in lung function	Change from baseline in QOL
Bachert et al,^36^ 2016	RCT (NCT01920893)	Severe CRSwNP refractory to intranasal CSs (n = 60)	58%/100%	FEV_1_∗ +200 mL (95% CI, −20 to 500) after 16 wk (*P* = .07)	SNOT-22 score Baseline: 41.4 ± 18.2 −18.1 points (95% CI, −25.6 to −10.6) after 16 wk (*P* < .001)
Bachert et al,^16^ 2019	RCT (LIBERTY NP SINUS-24 and SINUS-52)	Severe uncontrolled CRSwNP refractory to intranasal CS (n = 724)	59%/100%	FEV_1_† Baseline: 2.6 L +210 mL (95% CI, 130 to 290) after 24 wk (*P* < .0001)	SNOT-22 score Baseline: 50.9 ± 20.7 −21.1 (−25.2 to −17.1) after 24 wk, and −17.4 (−20.9 to −13.9) after 52 wk (*P* < .0001)
Bachert et al,^37^ 2020	*Post hoc* (NCT01920893)	Severe CRSwNP refractory to intranasal CSs (n = 60)	58%/100%	NA	SNOT-22 score∗ Baseline: 41.4 ± 18.2 −18.1 (95% CI, −25.6 to −10.6) after 16 wk (*P* < .001)
Bertlich et al,^46^ 2021	OBS retrospective	N-ERD (n = 31)	100%/100%	NA	SNOT-22 score 68.1 ± 13.9 at baseline to 20.1 ± 13.9 after 6.4 ± 2.7 mo (*P* < .001)
Maspero et al,^42^ 2020	*Post hoc* (LIBERTY ASTHMA QUEST)	Uncontrolled moderate to severe asthma (n = 1902)	100%/20%	Pre-BD FEV_1_† Baseline: 1.9 ± 0.6 L (200 mg)/1.7 ± 0.5 (300 mg) +280 mL (95% CI, 150 to 410) with the 200-mg dose (*P* < .0001) and +160 mL (30 to 280) with the 300-mg dose (*P* = .02) at week 52	SNOT-22 score∗† Baseline: 41.3 ± 18.0 (200 mg)/42.8 ± 18.0 (300 mg) −11.9 (−17.6 to −6.2) for dupilumab 200 mg (*P* < .0001) and −10.3 (−15.8 to −4.9) for dupilumab 300 mg (*P* = .0002) after 52 wk
Fujieda et al,^38^ 2021	*Post hoc* (LIBERTY NP SINUS-52)	Severe CRSwNP refractory to intranasal CS (n = 45)	63%/100%	FEV_1_† Baseline: 2.0 ± 0.5 L in arm A, 2.0 ± 0.6 L in arm B‡ +340 mL (95% CI, 50 to 630) after 24 wk (*P* = .02)	SNOT 22 score Arm A‡: −16.1 (−25.8 to −6.5) at 24 wk (*P* = .001), −18.9 (−29.1 to −8.8) at 52 wk (*P* = .02) Arm B‡: −11.4 (−20.8 to −1.9) at 24 wk (*P* = .0002), −11.5 (−21.4 to −1.6) at 52 wk (*P* = .02)
Hopkins et al,^40^ 2021	*Post hoc* (LIBERTY NP SINUS-24 and SINUS-52)	Severe CRSwNP refractory to intranasal CS (n = 724)	59%/100%	NA	SNOT-22 score Baseline value (range, 49.7 to 52, depending on the number of previous surgeries) significantly improved (≥8.9) at week 24
Laidlaw et al,^1^ 2021	RCT pool analysis (LIBERTY NP SINUS-24 and SINUS-52)	Severe CRSwNP refractory to intranasal CSs (n = 724)	59%/100%	FEV_1_† Baseline: 2.6 ± 0.9 L +210 mL (95% CI, 130 to 290) after 24 wk (*P* < .001)	SNOT-22 score† Baseline: 52.2 ± 19.8 −30.6 after 24 wk (*P* < .001)
Pelaia et al,^48^ 2021	OBS retrospective	Severe asthma(n = 20)	100%/100%	Pre-BD FEV_1_∗† 2.0 ± 0.9 L at baseline to 2.3 ± 1.0 L at week 4 (*P* < .01)	SNOT-22 score† 58.3 ± 21.6 at baseline to 18.9 ± 16.5 after 4 wk (*P* < .0001)
Buchheit et al,^45^ 2022	Open-label trial	N-ERD (n = 22)	100%/100%	FEV_1_∗† FEV_1_% at baseline (75.7 ± 19.6) improved at week 4 (12.6%, *P* = .0002) and 12 (12.1%, *P* = .002)	SNOT-22 score† Baseline: 48.7 ± 22.3 −34.4 points at week 4 (*P* < .0001), sustained after 12 wk
Dharmarajan et al,^47^ 2022	OBS retrospective	CRSwNP (n = 108)	74%-87%/100%	NA	SNOT-22 score 38.2 ± 21.0 to 23.8 ± 18.2 (NS) Significant improvement vs FESS in extranasal rhinologic (*P* = .02) and olfaction (*P* = .04) domains after 12.2 mo
Fujieda et al,^39^ 2022	*Post hoc* (LIBERTY NP SINUS-52)	Severe CRSwNP refractory to intranasal CSs (n = 438)	63%/100%	NA	SNOT-22 score Baseline: 53.1 ± 20.4 −19.2 (−23.5 to −14.8) at week 24, −23.9 (−28.2 to −19.7) at week 52 (*P* ≤ .027)
Lee et al,^41^ 2022	*Post hoc* (LIBERTY NP SINUS-24 and SINUS-52)	Severe CRSwNP refractory to intranasal CSs (n = 724)	59%/100%	NA	SNOT-22 score Baseline: 50.9 ± 20.7 −36.6% (−41.9% to – 31.3%) at week 24 (*P* < .0001)
Mullol et al,^43^ 2022	*Post hoc* (LIBERTY NP SINUS- 24 and SINUS- 52)	Severe CRSwNP refractory to intranasal CSs (n = 724)	59%/100% 28% N-ERD	FEV_1_§ Baseline: 2.6 ± 0.9 +260 mL (95% CI, 150 to 360) in patients with AERD after 24 wk (*P* < .0001)	SNOT-22 score§ Baseline: 52.9 ± 19.6 −24.4 (−29.5 to −19.2) in patients with AERD after 24 wk (*P* < .0001)

SNOT-22 scores range from 0 to 110. Higher SNOT-22 total-scores indicate worse symptoms and worse QOL. AQLQ scores range from 1 to 7. Higher AQLQ scores indicate better QOL.

*CS*, Corticosteroid; *FESS*, functional endoscopic sinus surgery; *NA*, not assessed; *N-ERD*, NSAID-exacerbated respiratory disease; *NS*, not significant; *NSAID*, nonsteroidal anti-inflammatory drug; *OBS*, observational study; *post-BD*, postbronchodilator; *pre-BD*, prebronchodilator.

∗ This study reports other lung function and QOL parameters.

† Results for patients with both asthma and CRSwNP are presented.

‡ Arm A: dupilumab 300 mg every 2 wk for 52 wk; arm B: dupilumab 300 mg every 2 wk for 24 wk followed by every 4 wk for 28 wk.

§ Results in the population with AERD (CRSwNP and 89% asthma).

An observational study showed a significant increase in FEV_1_ (300 mL) and a decrease in SNOT-22 scores (39.4 points) in patients with UAD after only 4 weeks.^48^ The first RCT evaluating dupilumab in patients with severe CRSwNP (58% patients with asthma) reported a numerical FEV_1_ increase (200 mL) and a significant reduction (18.1 points) in SNOT-22 scores after 16 weeks.^36^^,^^37^ Dupilumab consistently improved both lung function and QOL in the LIBERTY NP SINUS-24 and SINUS-52 populations (patients with severe CRSwNP, 59% patients with asthma). In the RCT, FEV_1_ significantly increased (210 mL) after 24 weeks, and the SNOT-22 score significantly decreased by 21.1 and 17.4 points after 24 and 52 weeks, respectively, in the subgroup of patients with CRSwNP and asthma.^16^ These improvements were further confirmed in subsequent *post hoc* analyses.^38^^,^^44^ Additional analyses evaluated the impact of dupilumab on the QOL of the overall CRSwNP population. Two of them showed consistent improvements in the SNOT-22 score after 24 weeks (19.2 points^39^ and 36.6%^41^) and 52 weeks (23.9 points),^39^ and another showed that the SNOT-22 score significantly improved 1 or more minimal clinically important difference at week 24, regardless of the number of previous surgeries.^40^

QOL has been shown to improve in patients with CRSwNP (74%-87% patients with asthma) treated with dupilumab versus functional endoscopic sinus surgery.^47^ However, the *post hoc* analysis of the liberty ASTHMA QUEST study revealed a significant increase in FEV_1_ (160-280 mL) and decrease in SNOT-22 scores (10.3-11.9 points) in patients with asthma with CRSwNP (20%) who received dupilumab for 52 weeks.^42^

In patients with AERD, the SNOT-22 score decreased by 48.0 points after at least 6 months, by 34.4 points at week 4, and by 24.4 points after 24 weeks in an observational study, open-label trial, and *post hoc* analysis, respectively.^43^^,^^45^^,^^46^ Notably, baseline SNOT-22 values varied across studies, and were particularly high in Bertlich et al.^46^ Lung function was shown to significantly improve after 4 and 12 weeks in the open-label trial (12%),^45^ and at week 24 in the *post hoc* analysis (260 mL).^43^

### Mepolizumab

A total of 10 studies reported outcomes with mepolizumab: 2 RCTs,^17^^,^^49^ 1 open-label trial,^50^ and 7 observational studies.^28^^,^^51^, ^52^, ^53^, ^54^, ^55^, ^56^ Asthma was the primary diagnosis in 8 studies and CRSwNP in 2 studies (Table III). Only 2 observational studies, 1 retrospective^56^ and 1 prospective,^54^ and the open-label trial^50^ reported outcomes in 100% patients with UAD; the remaining studies included 68% to 78% patients with asthma in the population with CRSwNP,^17^^,^^49^ and 34% to 88% patients with CRSwNP in the population with asthma.^28^^,^^51^, ^52^, ^53^^,^^55^ Eight studies included data on QOL and 7 on lung function outcomes; 5 of them reported both.^49^^,^^50^^,^^52^^,^^54^^,^^55^

**Table III:** Studies reporting lung function and/or QOL with mepolizumab and reslizumab

Reference	Study design	Population (n)	Asthma/CRSwNP	Change from baseline in lung function	Change from baseline in QOL
**Mepolizumab**					
Bachert et al,^49^ 2017	RCT	Severe CRSwNP requiring surgery (n = 107)	78%/100%	FEV_1_∗ Baseline: 3.2 ± 1.0 L +160 mL (95% CI, −20 to 340) at week 25 (*P* = .077)	SNOT-22 score Baseline: 51.5 ± 17.0 −13.2 (95% CI, −22.2 to −4.2) at week 25 (*P* < .005)
Kurosawa et al,^50^ 2019	Open-label trial	Severe eosinophilic asthma (n = 11)	100%/100% 55% N-ERD	FEV_1_† FEV_1_%: 69.0% ± 10.5% at baseline to 73.3% ± 8.4% at week 24 and 73.9% ± 8.8% at week 48 (both *P* < .05)	SNOT-22 score† decreased by 18.0 points (*P* < .01) at week 48
Bandi et al,^28^ 2020	OBS prospective	Patients with severe asthma (n = 40)	100%/85%	NA	SNOT-22 score†‡ 64.5 (IQR, 42.7 to 80.5) at baseline to 37.5 (10.5 to 55.5) at 52 weeks (*P* = .002)
Cameli et al,^52^ 2020	OBS retrospective	Severe eosinophilic asthma (n = 27)	100%/88%	FEV_1_∗ Post-BD FEV_1:_ 2.5 ± 0.9 L at baseline to 2.8 ± 1.0 L at week 4 and 2.9 ± 1.0 L at week 24 (*P* = .028)	SNOT-22 score 40.5 ± 21.9 at baseline to 21.6 ± 13.2 at week 4 and 23.6 ± 13.2 at week 24 (*P* = .018)
Crimi et al,^53^ 2020	OBS retrospective	Severe refractory eosinophilic asthma and multiple comorbidities (n = 31)	100%/77%	FEV_1_∗ 2.1 ± 0.7 L at baseline to 2.3 ± 0.7 L after 12 mo (*P* = .02)	NA
Harvey et al,^55^ 2020	OBS retrospectiv/prospective	Severe eosinophilic asthma (n = 309)	100%/34%	FEV_1_ FEV_1_% predicted in patients with high vs low eosinophil level§: 8.0 (IQR, −0.9 to 16.3) vs 3.2 (−1.7 to 8.8) after 12 mo (*P* = .032)	AQLQ score improvement in patients with high vs low eosinophil level§: 1.4 ± 1.2 vs 1.0 ± 1.1 at week 12 (*P* = .019); 1.6 ± 1.3 vs 1.1 ± 1.1 at week 24 (*P* = .026)
Yilmaz et al,^56^ 2020	OBS retrospective	OCS-dependent severe eosinophilic asthma (n = 16)	100%/100%	FEV_1_† FEV_1_% 80% ± 30.7% at baseline to 84% ± 26% at week 12 (*P* = .342), and 84.6% ± 26% at week 24 (*P* = .392)	NA
Bajpai et al,^51^ 2021	OBS retrospective	Asthma and CRSwNP (n = 247)	100%/73%	NA	SNOT-22 score†|| Baseline: 42.6 (95% CI, 36.2 to 49.0) −17.3 (95% CI, −25.0 to −9.6) at longest follow-up (>12 mo) (*P* < .001)
Detoraki et al,^54^ 2021	OBS prospective	Severe eosinophilic asthma (n = 44)	100%/100%	FEV_1_† FEV_1_% 68.1% ± 22.8% at baseline to 77.4% ± 22.5% at week 24 (*P* = .295), and 82.1% ± 22.5% at week 52 (*P* = .044)	SNOT-22 score† 51.5 ± 21.2 at baseline to 31.7 ± 17.4 at week 24 (*P* < .001), and 29.7 ± 21.5 at week 52 (*P* < .001)
Han et al,^17^ 2021	RCT (SYNAPSE)	Severe CRSwNP (n = 407)	68%-74%/100%	NA	SNOT-22 score Baseline: 63.7 ± 17.6 –16.5 (–23.6 to –9.4) at week 52 (*P* = .0032)
**Reslizumab**					
Castro et al,^57^ 2011	RCT	Poorly controlled eosinophilic asthma (n = 106)	100%/30%-42%	FEV_1_∗ Baseline: 2.1 ± 0.6 L +199 mL (−11 to 409) after 15 wk (*P* = .063)	NA
Weinstein et al,^21^ 2019	*Post hoc* (BREATH phase 3 trials)	Eosinophilic asthma with self-reported CRS (n = 953)	100%/16%	FEV_1_† Baseline: 2.0 ± 0.7 L +327 mL after 52 wk (*P* < .001)	AQLQ score† Baseline: 4.1 ± 1.1 0.67 (0.4 to 1.0) after 52 wk (*P* < .001)

SNOT-22 scores range from 0 to 110. Higher SNOT-22 total-scores indicate worse symptoms and worse QOL. AQLQ scores range from 1 to 7. Higher AQLQ scores indicate better QOL.

*CRS*, Chronic rhinosinusitis; *IQR*, interquartile range; *NA*, Not assessed; *N-ERD*, NSAID-exacerbated respiratory disease; *NSAID*, nonsteroidal anti-inflammatory drug; *OBS*, observational study; *OCS*, oral corticosteroid; *post-BD*, postbronchodilator.

∗ This study reports other lung function and QOL parameters.

† Results for patients with both asthma and CRSwNP are presented.

‡ Results of the 20 patients who received mepolizumab are presented.

§ High and low eosinophil levels defined as >600 cells/μL and ≤600 cells/μL, respectively.

|| Results of the 115 patients who received anti–IL-5 biologics (mostly mepolizumab) are presented.

In the 3 mepolizumab studies in patients with UAD, FEV_1_ increases were statistically significant in the open-label trial (at week 24 and 48)^50^ and in 1 observational study (at week 52),^54^ but only numerical in patients dependent on oral corticosteroid.^56^ SNOT-22 scores significantly decreased by 18.0 points at week 48,^50^ by 19.8 points at week 24, and by 21.8 points at week 52.^54^ A significant improvement in QOL was reported in the population with CRSwNP in the 2 observational studies in patients with asthma. With similar follow-up (≥52 weeks), the SNOT-22 score decreased by 17.3 and 27.0 points in the retrospective^51^ and prospective studies,^28^ respectively. Of note, the baseline SNOT-22 value was lower in the former and sample size was smaller in the latter.

Mepolizumab was associated with significant FEV_1_ increases in patients with severe eosinophilic asthma and CRSwNP (80%-90%). In 2 observational studies with similar baseline values, FEV_1_ improved after 4 weeks (300 mL) and 24 weeks (400 mL),^52^ and 12 months (200 mL),^53^ respectively. A significant reduction of 17 to 19 points in the SNOT-22 score was also observed in the former study.^52^ The study of Harvey et al,^55^ which included 34% of patients with CRSwNP, reported substantial improvements in FEV_1_ (after 12 months) and AQLQ score (at 12 and 24 weeks) in patients with high (>600 cells/μL) versus low eosinophils level.^55^ Mepolizumab treatment resulted in QOL improvement in 2 RCTs that included patients with severe CRSwNP (68%-78% patients with asthma). SNOT-22 scores significantly decreased by 13.2 points after 25 weeks^49^ and by 16.5 points after 52 weeks^17^; the baseline value was lower in the former study. A FEV_1_ numerical increase of 160 mL at week 25 was also reported.^49^

### Reslizumab

Only 2 studies reported outcomes with reslizumab: 1 RCT^57^ and 1 *post hoc* analysis.^21^ In both studies, asthma was the primary diagnosis, and 30% to 42% and 16% of patients had CRSwNP, respectively (Table III). With similar baseline FEV_1_ values, increases of nearly 200 mL after 15 weeks and 327 mL after 52 weeks were reported in the overall population of the RCT and in the population with self-reported CRSwNP in the *post hoc* analysis, respectively.^21^^,^^57^ A significant improvement in AQLQ score was shown in the latter.

### Omalizumab

Of the 13 studies reporting outcomes with omalizumab, 2 were RCTs,^18^^,^^58^ 1 was an open-label extension RCT,^59^ 2 were *post hoc* analyses,^60^^,^^61^ and 8 were observational studies.^28^^,^^51^^,^^62^, ^63^, ^64^, ^65^, ^66^, ^67^ Overall, 6 studies analyzed asthma, 5 CRSwNP, and 2 AERD as the primary diagnosis of the study populations (Table IV). One RCT^58^ and 4 observational studies (2 prospective^63^^,^^66^ and 2 retrospective)^62^^,^^67^ reported data on 100% patients with UAD, whereas the remaining studies included 49% to 61% patients with asthma in the population with CRSwNP,^18^^,^^60^ and 14% to 82% patients with CRSwNP in the population with asthma.^28^^,^^51^^,^^61^ Five studies reported lung function and 11 QOL outcomes; of these, 3 studies included both.^64^, ^65^, ^66^

**Table IV:** Studies reporting lung function and/or QOL with omalizumab

Reference	Study design	Population (n)	Asthma/CRSwNP	Change from baseline in lung function	Change from baseline in QOL
Gevaert et al,^58^ 2013	RCT	CRSwNP with asthma (n = 24)	100%/100%	NA	AQLQ score∗† Baseline: 5.8 (5.4 to 6.4) 0.81 points after 16 wk (*P* = .003)
Bidder et al,^63^ 2018	OBS prospective	Severe allergic asthma (n = 13)	100%/100%	NA	SNOT-22 score∗ 52.0 (range, 27 to 78) at baseline to 24.5 (1 to 42) at 4 wk (*P* = .003) and 30 (3 to 60) at 16 wk (*P* = .009)
Bandi et al,^28^ 2020	OBS prospective	Patients with severe asthma (n = 40)	100%/82%	NA	SNOT-22 score∗‡ 48.0 (IQR, 33 to 78.5) at baseline to 22.5 (12 to 33.5) at 52 wk (*P* = .047)
Cameli et al,^52^ 2020	OBS retrospective	N-ERD (n = 8)	100%/100%	FEV_1_† 2.3 ± 0.9 L at baseline to 2.6 ± 0.9 L after 12 mo (*P* = .016)	SNOT-22 score 29 ± 8.8 at baseline, improved after 12 mo (*P* = .03)
Forster-Ruhrmann et al,^65^ 2020	OBS retrospective	N-ERD (n = 16)	100%/100%	FEV_1_ improved from 80% at baseline to 89% after 9 mo (*P* = .04)	RSOM-31 7.8 at baseline to 4.1 at 12 wk (*P* < .001), 3.9 at 24 wk (*P* < .001), and 3.6 at 9 mo (*P* < .05)
Gevaert et al,^18^ 2020	RCT (POLYP 1, POLYP 2)	CRSwNP (n = 265)	49%-61%/100%	NA	SNOT-22 score† Baseline: 59.2 ± 20.5 (POLYP 1)/60.5 ± 15.3 (POLYP 2) –16.1 (–21.9 to –10.4) in POLYP 1, –15.0 (–21.3 to –8.8) in POLYP 2 (both *P* < .0001) at 24 wk
Heffler et al,^61^ 2020	*Post hoc* (PROXIMA)	Severe allergic asthma (n = 123)	100%/14%	FEV_1_∗† Baseline: 1.7 ± 0.8 L +7.42% (0.44 to 35.00) in % of predicted FEV_1_ at 12 mo (*P* = .005)	NA
Ruiz-Hornillos et al,^66^ 2020	OBS prospective	Moderate to severe persistent allergic asthma (n = 16)	100%/100%	FEV_1_∗ FEV_1_% 74.0 (IQR, 59.3 to 82.8) at baseline to 83.0 (69.3 to 94.5) after 52 wk (*P* = .026)	Mini-AQLQ score∗† 62.0 (IQR, 37.0 to 75.0) at baseline to 61.0 (47.5 to 92.5) after 52 wk (*P* = .136)
Tiotiu et al,^67^ 2020	OBS retrospective	Severe allergic asthma (n = 24)	100%/100%	FEV_1_∗† FEV_1_% 60.1% ± 18.2% at baseline to 72.9% ± 19.4% after 24 wk (*P* < .001)	NA
Armengot-Carceller et al,^62^ 2021	OBS retrospective	Recalcitrant CRSwNP and mild asthma (n = 23)	100%/100%	NA	SNOT-22∗ score 59.0 ± 25.4 at baseline to 24.9 ± 20.1 at 6 mo, and 19.7 ± 19.6 at 12 mo
Bajpai et al,^51^ 2021	OBS retrospective	Asthma and CRSwNP (n = 247)	100%/73%	NA	SNOT-22 score§ Baseline: 42.5 (95% CI, 28.5 to 56.4) −18.1 (95% CI, −42.6 to 6.3) at longest follow-up (>12 mo) (*P* = .109)
Damask et al,^60^ 2022	*Post hoc* (POLYP 1, POLYP 2)	CRSwNP (n = 265)	54%-60%/100%	NA	SNOT-22 score 59.5 ± 20.0 at baseline, improved after 24 wk
Gevaert et al,^59^ 2022	RCT open-label extension (POLYP 1, POLYP 2)	CRSwNP (n = 249)	54%-60%/100%	NA	SNOT-22 score† Baseline (week 24): 36.4 ± 23.5 −6.1 (95% CI, −10.3 to 0.9) at week 52 (*P* < .0056)

*IQR*, Interquartile range; *NA*, not assessed; *N-ERD*, NSAID-exacerbated respiratory disease; *NSAID*, nonsteroidal anti-inflammatory drug; *OBS*, observational study; *post-BD*, postbronchodilator; *pre-BD*, prebronchodilator; *RSDI*, Rhinosinusitis Disability Index; *RSOM-31*, Rhinosinusitis Outcome Measure.

∗ Results for patients with both asthma and CRSwNP are presented.

† This study reports other lung function and QOL parameters.

‡ Results of the 11 patients who received omalizumab are presented.

§ SNOT-22 scores range from 0 to 110. Higher SNOT-22 total-scores indicate worse symptoms and worse QOL. AQLQ scores range from 1 to 7. Higher AQLQ scores indicate better QOL.

Observational studies reported significant increases in FEV^1^ after 52 weeks^66^ and 24 weeks^67^ in patients with UAD treated with omalizumab. QOL improvement was associated with a significant increase in the AQLQ score (0.8 points)^58^ or reduction in the SNOT-22 score (22.0 points) after 16 weeks.^63^ A decrease in the SNOT-22 score over time was also observed (34.1 and 39.3 points at 24 and 52 weeks, respectively).^62^ In the 2 studies with patients with severe asthma analyzing the population with CRSwNP, significant improvements in lung function^61^ and QOL were observed after 52 weeks.^28^

The publications associated with POLYP 1 and POLYP 2 trials reported a significant decrease in the SNOT-22 score at week 24 (15.0-16.1 points in the RCT)^18^^,^^60^ and week 52 (6.1 points [from the 24-week assessment] in the open-label extension)^59^ in the population with CRSwNP. In contrast, the reduction in the SNOT-22 score was only numerical in an observational study of patients with asthma with CRSwNP.^51^ In patients with AERD, 2 observational studies reported significant increases in FEV_1_ after 9 and 12 months, as well as improved QOL.^64^^,^^65^

## Discussion

This systematic review provides an updated overview of the role of biologics in lung function and QOL of patients with asthma and CRSwNP from the comprehensive perspective of UAD. Our search retrieved 48 original studies (11 RCTs, 11 *post hoc* analyses, 3 open-label/extension trials, and 23 observational studies) with an overall moderate to high methodological quality. In total, 18 studies reported data on QOL, 8 on lung function, and 22 on both outcomes. Outcomes of benralizumab and reslizumab were mostly identified in populations with asthma, whereas CRSwNP was predominant in dupilumab and mepolizumab studies, and omalizumab was evenly distributed across populations. Significant improvements in FEV_1_ and/or the SNOT-22 score have been described in patients with asthma and CRSwNP and/or AERD who received benralizumab (12 studies), dupilumab (14 studies), mepolizumab (10 studies), omalizumab (13 studies), and reslizumab (2 studies).

Patient populations in terms of multimorbidity (ie, percentage with asthma and CRSwNP) were highly heterogeneous across studies. The primary diagnosis was asthma, CRSwNP, or AERD in 25 studies, 19 studies, and 4 studies, respectively. In asthma studies, most of which were observational and retrospective, the percentage of patients with CRSwNP varied from 14% to 88%. Most studies in patients with CRSwNP, however, were RCTs and *post hoc* analyses, and included a higher proportion of patients with asthma (range, 55%-92%). Overall, 31% of selected studies (15 of 48) included 100% populations with UAD, although most of them were performed in patients with asthma. Lung function and/or QOL outcomes in patients with UAD were often found in small observational retrospective studies, which highlights the need for clinical trials and larger real-world studies focusing on the UAD concept rather than the comorbidity.

Biologics have shown promise in the management of UAD.^2^^,^^68^ Five biologics have been approved for severe asthma, among which dupilumab, omalizumab, and mepolizumab were also approved for the treatment of severe CRSwNP. These agents reduce exacerbation rates and the daily oral corticosteroid dose, and also improve asthma control and lung function.^16^^,^^50^^,^^55^^,^^61^ Biologics also improve QOL by ameliorating nasal symptoms associated with CRSwNP, such as loss of smell, and reduce the need for systemic corticosteroids and endoscopic sinus surgery.^16^^,^^24^^,^^49^^,^^58^ Our study shows that treating patients with UAD with benralizumab, dupilumab, mepolizumab, or omalizumab for at least 24 weeks significantly improves FEV_1_ and/or the SNOT-22 score.

Although FEV_1_ is currently one of the criterion standards for assessing lung function,^19^ several other methods were described in the studies retrieved, such as predicted FEV_1_ and prebronchodilator or postbronchodilator FEV_1_. Furthermore, different measurement time points were used (ie, from week 4 to week 52), week 24 being the most frequent. Benralizumab and dupilumab high-quality RCTs specifically reported FEV_1_ improvements in patients with asthma and CRSwNP, which was further confirmed in mepolizumab and omalizumab *post hoc* analyses and observational studies. However, the severity of asthma and CRSwNP, when considered as a comorbid condition, was rarely reported. We observed that clinical trials and real-world studies usually analyze 1 primary disease (severe asthma or severe CRSwNP) plus a comorbid condition. Furthermore, the diagnosis of CRSwNP in studies in patients with severe asthma is based on clinical history, whereas the severity of asthma or its treatment is not commonly reported in CRSwNP studies.

It has been described that UAD negatively impacts QOL^7^ and these patients have poorer outcomes than those reported in patients with asthma or CRSwNP. Our results showed that QOL in patients with asthma and CRSwNP is frequently measured using the SNOT-22 (35 studies), AQLQ (7 studies), and/or St. George’s Respiratory questionnaire (2 studies) scales. Of these, SNOT-22 was reported in most studies, even when asthma was the primary diagnosis. Noticeably, the baseline SNOT-22 score was lower in patients with asthma versus patients with CRSwNP. Significant reductions in the SNOT-22 score were observed across studies after at least 24 weeks of treatment with benralizumab, dupilumab, mepolizumab, or omalizumab, with subsequent improvements in QOL that persisted or increased in the long-term. Although some small observational studies reported greater reductions in SNOT-22 scores, these data need to be confirmed in larger populations.

Given the importance of including both lung function and QOL among the main outcomes of studies in patients with asthma and CRSwNP, future research could focus on analyzing the potential correlation between these outcomes. In total, 22 of the 48 studies evaluated the role of biologics (benralizumab [5], dupilumab [8], mepolizumab [5], omalizumab [3], and reslizumab [1]) in both outcomes. Although some authors acknowledged a potential association between lung function and QOL,^38^^,^^42^^,^^44^^,^^54^ none of the studies included in this review analyzed this phenomenon.

Our systematic review had some limitations. Some studies were not designed for UAD analysis because data from CRSwNP were primarily obtained from the clinical history. However, most RCTs including patients with CRSwNP performed a prospective group analysis of populations with or without asthma and, in some cases (eg, dupilumab studies), a statistical comparison is shown. Second, although most studies were observational and/or performed in small populations, results were in line with data observed in the RCTs. Third, analysis of the evidence was challenging due to the high heterogeneity of patient populations and variables across studies, which prevented us from making reliable comparisons. In fact, the effect size was different among some of the included studies, even as to the same end point. Therefore, data should be interpreted with caution. Lastly, although the search strategies were design to find all available UAD evidence, studies that were not classified as such might have been overlooked.

### Conclusions

We systematically reviewed the latest evidence on the effect of biologics on lung function and QOL in patients with UAD, focusing on severe asthma and severe CRSwNP and/or AERD. In these patient populations, benralizumab, dupilumab, mepolizumab, omalizumab, and reslizumab led to an overall improvement in lung function and QOL. The primary diagnosis in most studies was severe asthma or severe CRSwNP; only 15 studies included 100% patients with UAD. Our results showed the high heterogeneity of populations, scores, measurements, and time points, thereby highlighting the need for unified criteria that will allow researchers to compare data and draw reliable conclusions. Further studies will provide an in-depth understanding of the baseline characteristics of patients with multimorbid conditions and allow a more comprehensive evaluation of the effect of biologics in both diseases under the UAD concept.

## Disclosure statement

This work was funded by Sanofi. Editorial assistance was provided by Celia Miguel-Blanco (Medical Science Consulting; Valencia, Spain). The authors were not compensated for writing the manuscript. The sponsor was not involved in the protocol design, data analysis, or manuscript writing.

Disclosure of potential conflict of interest: J. Domínguez-Ortega has received funding for research, and honoraria for consultancy and conferences from 10.13039/100004325AstraZeneca, 10.13039/100019719Chiesi, and 10.13039/100004330GlaxoSmithKline (GSK); honoraria for consultancy and conferences from Bial, 10.13039/100004336Novartis, 10.13039/100004339Sanofi, and Teva; and speaker fees from ALK, LETI Pharma, and Mundipharma. J. Mullol is a member of national or international advisory boards and has received speaker fees or funding for clinical trials and research projects from Allakos, AstraZeneca, Genentech, GSK, Glenmark, Menarini, Mitsubishi-Tanabe, MSD, Viatris/MEDA Pharma, Novartis, Proctor & Gamble, Regeneron Pharmaceuticals, Inc, Sanofi, UCB Pharma, and Noucor/Uriach Group. F. J. Álvarez Gutiérrez has participated in speaking activities and advisory boards, and has provided consultancy services sponsored by AstraZeneca, ALK, Bial, Boehringer-Ingelheim, Chiesi, GSK, Mundipharma, Novartis, Orion-Pharma, and Sanofi from 2017 to 2022. J. A. Castillo has received honoraria for lectures or courses, and research grants from MSD, AstraZeneca, Boehringer-Ingelheim, Uriach, GSK, Leti, and ALK. J. M. Olaguibel has received honoraria for consultancy from ALK, AstraZeneca, and Eversens; industry-sponsored grants from Sanofi and Eversens; lecture fees from GSK, Chiesi, MSD, AstraZeneca, and Mundipharma; and belongs to the editorial board of *the Journal of Investigational Allergology and Clinical Immunology*. M. Blanco-Aparicio has received honoraria for lectures, courses, participation in monographs and standards, and scientific advice from AstraZeneca, Sanofi, Esteve, GSK, Menarini, Novartis, and TEVA. C. Miguel-Blanco declares no conflict of interest.

Data Availability: The data supporting the findings of this study are available in the article and its Online Repository material. Additional data generated during the systematic review are available from the corresponding author (M.B.A.) on request.

**Key messages**

• **We reviewed the available evidence on the effect of biologics on the UAD, namely, asthma and CRSwNP.**
• **Despite the heterogeneity of populations and outcome measurements, biologics consistently improved lung function and QOL in patients with asthma and CRSwNP.**
• **This could guide treatment decisions in these patients from the UAD approach, thereby emphasizing a comprehensive management of upper and lower respiratory tract diseases.**
