# Supplementary material for: The effect of biologics in lung function and quality of life of patients with united airways disease: A systematic review
Source: J Allergy Clin Immunol Glob. 2023 Sep 28;3(1):100174. doi: 10.1016/j.jacig.2023.100174 (PMC10616425; doi:10.1016/j.jacig.2023.100174)
Supplement: Supplementary data [file mmc1.docx]

**SUPPLEMENTARY MATERIAL**

**Table S1.** PRISMA checklist.

| **Section and Topic** | **Item #** | **Checklist item** | **Location where item is reported** |
| --- | --- | --- | --- |
| **TITLE** | | |  |
| Title | 1 | Identify the report as a systematic review. | 1 |
| **ABSTRACT** | | |  |
| Abstract | 2 | See the PRISMA 2020 for Abstracts checklist. | 4 |
| **INTRODUCTION** | | |  |
| Rationale | 3 | Describe the rationale for the review in the context of existing knowledge. | 7-8 |
| Objectives | 4 | Provide an explicit statement of the objective(s) or question(s) the review addresses. | 8 |
| **METHODS** | | |  |
| Eligibility criteria | 5 | Specify the inclusion and exclusion criteria for the review and how studies were grouped for the syntheses. | 8-9 |
| Information sources | 6 | Specify all databases, registers, websites, organisations, reference lists and other sources searched or consulted to identify studies. Specify the date when each source was last searched or consulted. | 9 |
| Search strategy | 7 | Present the full search strategies for all databases, registers and websites, including any filters and limits used. | 9, Table S2 |
| Selection process | 8 | Specify the methods used to decide whether a study met the inclusion criteria of the review, including how many reviewers screened each record and each report retrieved, whether they worked independently, and if applicable, details of automation tools used in the process. | 9 |
| Data collection process | 9 | Specify the methods used to collect data from reports, including how many reviewers collected data from each report, whether they worked independently, any processes for obtaining or confirming data from study investigators, and if applicable, details of automation tools used in the process. | 9 |
| Data items | 10a | List and define all outcomes for which data were sought. Specify whether all results that were compatible with each outcome domain in each study were sought (e.g. for all measures, time points, analyses), and if not, the methods used to decide which results to collect. | 8-9 |
|  | 10b | List and define all other variables for which data were sought (e.g. participant and intervention characteristics, funding sources). Describe any assumptions made about any missing or unclear information. | 8-9 |
| Study risk of bias assessment | 11 | Specify the methods used to assess risk of bias in the included studies, including details of the tool(s) used, how many reviewers assessed each study and whether they worked independently, and if applicable, details of automation tools used in the process. | 10 |
| Effect measures | 12 | Specify for each outcome the effect measure(s) (e.g. risk ratio, mean difference) used in the synthesis or presentation of results. | 10 |
| Synthesis methods | 13a | Describe the processes used to decide which studies were eligible for each synthesis (e.g. tabulating the study intervention characteristics and comparing against the planned groups for each synthesis (item #5)). | 10 |
|  | 13b | Describe any methods required to prepare the data for presentation or synthesis, such as handling of missing summary statistics, or data conversions. | 10 |
|  | 13c | Describe any methods used to tabulate or visually display results of individual studies and syntheses. | 10 |
|  | 13d | Describe any methods used to synthesize results and provide a rationale for the choice(s). If meta-analysis was performed, describe the model(s), method(s) to identify the presence and extent of statistical heterogeneity, and software package(s) used. | 10 |
|  | 13e | Describe any methods used to explore possible causes of heterogeneity among study results (e.g. subgroup analysis, meta-regression). | NA |
|  | 13f | Describe any sensitivity analyses conducted to assess robustness of the synthesized results. | NA |
| Reporting bias assessment | 14 | Describe any methods used to assess risk of bias due to missing results in a synthesis (arising from reporting biases). | NA |
| Certainty assessment | 15 | Describe any methods used to assess certainty (or confidence) in the body of evidence for an outcome. | NA |
| **RESULTS** | | |  |
| Study selection | 16a | Describe the results of the search and selection process, from the number of records identified in the search to the number of studies included in the review, ideally using a flow diagram. | 10, Figures S1 and S2 |
|  | 16b | Cite studies that might appear to meet the inclusion criteria, but which were excluded, and explain why they were excluded. | Figures S1 and S2 |
| Study characteristics | 17 | Cite each included study and present its characteristics. | 10-15 |
| Risk of bias in studies | 18 | Present assessments of risk of bias for each included study. | Table S3 |
| Results of individual studies | 19 | For all outcomes, present, for each study: (a) summary statistics for each group (where appropriate) and (b) an effect estimate and its precision (e.g. confidence/credible interval), ideally using structured tables or plots. | 10-15, tables 1, 2 3, and 4 |
| Results of syntheses | 20a | For each synthesis, briefly summarise the characteristics and risk of bias among contributing studies. | 10-15, tables 1-4, table S3 |
|  | 20b | Present results of all statistical syntheses conducted. If meta-analysis was done, present for each the summary estimate and its precision (e.g. confidence/credible interval) and measures of statistical heterogeneity. If comparing groups, describe the direction of the effect. | NA |
|  | 20c | Present results of all investigations of possible causes of heterogeneity among study results. | NA |
|  | 20d | Present results of all sensitivity analyses conducted to assess the robustness of the synthesized results. | NA |
| Reporting biases | 21 | Present assessments of risk of bias due to missing results (arising from reporting biases) for each synthesis assessed. | NA |
| Certainty of evidence | 22 | Present assessments of certainty (or confidence) in the body of evidence for each outcome assessed. | NA |
| **DISCUSSION** | | |  |
| Discussion | 23a | Provide a general interpretation of the results in the context of other evidence. | 15-18 |
|  | 23b | Discuss any limitations of the evidence included in the review. | 18 |
|  | 23c | Discuss any limitations of the review processes used. | 18 |
|  | 23d | Discuss implications of the results for practice, policy, and future research. | 15-17 |
| **OTHER INFORMATION** | | |  |
| Registration and protocol | 24a | Provide registration information for the review, including register name and registration number, or state that the review was not registered. | 8 |
|  | 24b | Indicate where the review protocol can be accessed, or state that a protocol was not prepared. | 8 |
|  | 24c | Describe and explain any amendments to information provided at registration or in the protocol. | NA |
| Support | 25 | Describe sources of financial or non-financial support for the review, and the role of the funders or sponsors in the review. | 2 |
| Competing interests | 26 | Declare any competing interests of review authors. | 2 |
| Availability of data, code and other materials | 27 | Report which of the following are publicly available and where they can be found: template data collection forms; data extracted from included studies; data used for all analyses; analytic code; any other materials used in the review. | 3 |

*From:* Page MJ, McKenzie JE, Bossuyt PM, Boutron I, Hoffmann TC, Mulrow CD, *et al*. The PRISMA 2020 statement: an updated guideline for reporting systematic reviews. BMJ 2021;372:n71. doi: 10.1136/bmj.n71

**Table S2.** Search terms and strategies.

| **Items** | **Search terms and synonyms** |
| --- | --- |
| #1 | (CRSwNP OR "chronic rhinosinusitis with nasal polyps" OR "chronic rhinosinusitis with nasal polyposis" OR "nasal polyp*" OR "nasal polyposis" OR NSAID-ERD OR “Samter’s triad” OR “aspirin-exacerbated respiratory disease”) AND (asthma OR asthmatic) |
| #2 | (antibod* OR biologic* OR "biological" OR "anti-IgE" OR omalizumab OR "anti-IL-5" OR mepolizumab OR reslizumab OR benralizumab OR "anti-IL-4" OR dupilumab) |
| #3 | (“quality of life” OR QOL OR HRQOL OR "health-related quality of life" OR "SNOT-22" OR "Sinonasal Outcome Test" OR "patient-reported outcomes" OR AQLQ OR "Asthma Quality of Life Questionnaire") |
| #4 | (FEV1 OR "forced expiratory volume in 1 second" OR spirometry OR ((lung OR pulmonary OR "respiratory") AND ("function" OR "capacity"))) |
|  | **MeSH for MEDLINE** |
| Asthma | Asthma[MeSH] |
| CRSwNP | Nasal Polyps[MeSH] |
| Biologics | Biological Therapy[MeSH] |
| Pulmonary function | Respiratory Function Tests[MeSH] |
| Quality of life | Quality of Life[MeSH] |
|  | **Search strategies** |
| Strategy 1 | #1 AND #2 AND #3 |
| Strategy 2 | #1 AND #2 AND #4 |
|  | **Filters** |
| Language | English and Spanish |
| Time | January 2010 - March 2022 |

**Table S3.** Quality assessment of studies selected for inclusion.

| **Study reference** | **Type of study** | **Quality assessment** | **CASP results^a^** | | |
| --- | --- | --- | --- | --- | --- |
|  |  |  | **Design** | **Methods** | **Outcomes** |
| Agache 2021 | Systematic review | high | +++ | +++ | +-+ |
| Armengot-Carceller 2021 | OBS retrospective | high | +++ | +-+ | +++ |
| Bachert 2019 | RCT | high | +++ | +++ | +++ |
| Bachert 2022 | RCT | high | +++ | +++ | +++ |
| Bachert 2020 | Post hoc analysis | high | +++ | +++ | +++ |
| Bachert 2016 | RCT | high | +++ | +++ | +++ |
| Bachert 2017 | RCT | high | +++ | +++ | +++ |
| Bagnasco 2020 | OBS retrospective | moderate-low | +++ | +-- | +-- |
| Bajpai 2021 | OBS retrospective | high | +++ | +-+ | +++ |
| Bandi 2020 | OBS prospective | high | +++ | +-+ | +-+ |
| Bertlich 2021 | OBS retrospective | moderate | +++ | +-- | +-+ |
| Bidder 2018 | OBS prospective | moderate-low | +++ | +-- | +-- |
| Buchheit 2022 | Open-label trial | high | +++ | +-+ | +++ |
| Cameli 2020 | OBS retrospective | high | +++ | +-+ | +-+ |
| Cameli 2020 | OBS retrospective | low | ++- | +-- | +-- |
| Canonica 2021 | Post hoc analysis | high | +++ | +++ | +++ |
| Castro 2011 | RCT | high | +++ | +++ | +++ |
| Chong 2020 | Systematic review | high | +++ | +++ | +-+ |
| Chong 2021 | Systematic review | high | +++ | +++ | +-+ |
| Crimi 2020 | OBS retrospective | high | +++ | +-+ | +++ |
| Damask 2022 | Post hoc analysis | high | +++ | +++ | ++- |
| Detoraki 2021 | OBS prospective | high | +++ | +-+ | +++ |
| Dharmarajan 2022 | OBS retrospective | high | +++ | +-- | +++ |
| Forster-Ruhrmann 2020 | OBS retrospective | moderate-high | +++ | +-+ | +-+ |
| Fujieda 2021 | Post hoc analysis | high | ++- | +++ | +++ |
| Fujieda 2022 | Post hoc analysis | high | +++ | +++ | +++ |
| Gevaert 2013 | RCT | high | +++ | +++ | +++ |
| Gevaert 2020 | RCT | high | +++ | +++ | +++ |
| Gevaert 2022 | open-label extension | moderate | +-+ | +-+ | +++ |
| Han 2021 | RCT | high | +++ | +++ | +++ |
| Harrison 2021 | RCT | high | +++ | +++ | +++ |
| Harvey 2020 | OBS retrospective /prospective | moderate-high | +++ | +-+ | +-+ |
| Heffler 2020 | Post hoc analysis | moderate | +++ | +++ | +-- |
| Hopkins 2021 | Post hoc analysis | moderate-high | ++- | +-+ | +++ |
| Iqbal 2020 | Systematic review | high | +++ | +++ | +-+ |
| Kurosawa 2019 | Prospective open-label trial | moderate | +++ | +-+ | +-- |
| Laidlaw 2021 | Pooled analysis RCT | moderate | ++- | +-+ | +++ |
| Lee 2022 | Post hoc analysis | moderate-high | ++- | +++ | ++- |
| Lombardo 2020 | OBS prospective | high | +++ | +-+ | +++ |
| Maspero 2020 | Post hoc analysis | high | +++ | +-+ | +++ |
| Matsuno 2020 | OBS retrospective | moderate-high | +++ | +-+ | +-+ |
| Menzella 2021 | OBS retrospective | moderate-high | +++ | +-+ | +-+ |
| Menzella 2020 | OBS retrospective | moderate-high | +++ | +-+ | +-+ |
| Mullol 2022 | Post hoc analysis | moderate | ++- | +-+ | +++ |
| Nolasco 2021 | OBS retrospective | high | +++ | +-+ | +++ |
| Numata 2020 | OBS retrospective | high | +++ | +-+ | +++ |
| Pelaia 2021 | OBS retrospective | moderate-high | +++ | +-+ | +-+ |
| Peters 2021 | Systematic review | high | +++ | +++ | +-+ |
| Rix 2015 | Systematic review | moderate-high | +++ | +++ | --+ |
| Ruiz-Hornillos 2020 | OBS prospective | moderate-high | +++ | +-+ | +-+ |
| Tiotiu 2020 | OBS retrospective | moderate-high | +++ | +-+ | +-+ |
| Tsetsos 2018 | Systematic review | moderate-high | +++ | +-+ | -++ |
| Tversky 2021 | RCT | high | +++ | +++ | +-+ |
| Weinstein 2019 | Post hoc analysis | moderate | ++- | +-+ | ++- |
| Wu 2021 | Systematic review | high | +++ | +++ | +-+ |
| Yilmaz 2020 | OBS retrospective | moderate | +++ | +-+ | +-- |

^a^Quality assessment was performed using CASP checklists for each type of study (<https://casp-uk.net/casp-tools-checklists/>). Results depicted in the table correspond to questions related to design (questions 1-3), methodology (questions 4-6) and outcomes (questions 7-9) in the corresponding checklists. Each positive (yes) response in the questionnaire is depicted as (+), otherwise it is indicated as (-). The increasing number of (+) indicates a greater quality assessment score. (Mullol J, et al. Management of United Airway Disease Focused on Patients With Asthma and Chronic Rhinosinusitis With Nasal Polyps: A Systematic Review. J Allergy Clin Immunol Pract. 2022 Sep;10(9):2438-2447.e9.)

OBS: observational study; RCT: randomized clinical trial.

**Figure S1.** PRISMA diagram for search strategy 1.

Records identified in databases

(*n* = 501)

**Screening**

**Selection**

Additional records identified from other sources
(*n* = 0)

Records after removing duplicates
(*n* = 290)

Screened records

(*n* = 290)

Records excluded by title/abstract
(*n* = 243):

Type of publication (*n* = 131)

Topic (*n* = 104)

Language (*n* = 8)

Full-text screening
(*n* = 47)

Records excluded and reasons for exclusion (*n* = 4):

Population (*n* = 2)

Out of topic outcomes (*n* = 2)

Records included for qualitative synthesis
(*n* = 43^‡^):

QoL (*n* = 17)

Lung function (*n* = 1)

Both (*n* = 17)

**Identification**

## cation

**Inclusion**

^‡^From the 43 included publications, 7 were systematic reviews.

**Figure S2.** PRISMA diagram for search strategy 2.

Records identified in databases

(*n* = 529)

**Screening**

**Selection**

Additional records identified from other sources
(*n* = 0)

Records after removing duplicates
(*n* = 323)

Screened records

(*n* = 323)

Records excluded by title/abstract
(*n* = 292):

Type of publication (*n* = 104)

Topic (*n* = 184)

Language (*n* = 4)

Full-text screening
(*n* = 31)

Records excluded and reasons for exclusion (*n* = 3):

Population (*n* = 2)

Out of topic outcomes (*n* = 1)

Records included for qualitative synthesis
(*n* = 28^‡^):

Lung function (*n* = 8)

QoL (*n* = 2)

Both (*n* = 17)

**Identification**

## cation

**Inclusion**

^‡^From the 28 included publications, 1 was a systematic review.
